# 

**Published:** 1883-12

**Authors:** 


*Bristol Medico-Chirurgical Journal.*

DECEMBER, 1883.

### CONTENTS.

ORIGINAL PAPERS:— *Page.*

Malarial Poisoning. By WM. JOHNSTON FYFFE, M.D. ... ... 143

A Record of Twenty-one Cases of Placenta Prævia. By JOSEPH GRIFFITHS SWAYNE, M.D. ... ... ... ... ... ... ... ... 166

An Epidemic of Tetanus. By ALFRED SHEEN, M.D. ... ... 173

A Study of "Medical Posture." By JOHN KENT SPENDER, M.D. ... ... ... ... ... ... ... ... ... ... ... ... ... 184

Abdominal Section in Perforating-Ulcer of the Stomach. A Suggestion. By NELSON C. DOBSON, F.R.C.S. ... ... 196

Retinoscopy. By ARTHUR B. PROWSE, M.D. ... ... ... ... 200

CLINICAL RECORDS:—

Cases of Muscular Atropy and Degeneration. By J. FENTON EVANS, M.B. ... ... ... ... ... ... ... ... ... ... 212

Note on the Utility of Iodoform in the Treatment of Phthisis, and its occasional Toxic Effects. By R. SHINGLETON SMITH, M.D., B.Sc., M.R.C.P. ... ... ... ... ... ... 218

A Case of Priapism. By A. H. BOYS, L.R.C.P. ... ... ... 224

Minute Hæmorrhages in the Pia Mater. By T. STEELE SHELDON, M.B. ... ... ... ... ... ... ... ... ... ... ... ... 225

Two Cases—One of Gouty Inflammation of the Testis, and another the Testicle of Mumps. By F. K. GREEN, F.R.C.S. ... ... ... ... ... ... ... ... ... ... ... ... 227

Successful Termination of a large Aneurism, without Surgical Interference. By HENRY WALDO, M.D. ... ... ... ... 232

UNIVERSITY COLLEGE, BRISTOL. MEDICAL SCHOOL:—

Address by DR. BEDDOE, F.R.S. Prize List ... ... ... ... 235

NOTES ON BOOKS ... ... ... ... ... ... ... ... ... ... ... ... ... 247

MEDICAL EXTRACTS ... ... ... ... ... ... ... ... ... ... ... ... 253

INDEX.

